# Supplementary material for: Patterns of microbial communities were shaped by bioavailable P along the elevation gradient of Shergyla Mountain, as determined by analysis of phospholipid fatty acids
Source: PLoS One. 2022 Jul 11;17(7):e0271101. doi: 10.1371/journal.pone.0271101 (PMC9273077; doi:10.1371/journal.pone.0271101)
Supplement: Attachment — Submitted filename: Comments_PONE-D-22-06081.docx [file pone.0271101.s002.docx]

**Review comments of ms # PONE_D_22_06081 entitled “Patterns of microbial communities were shaped by bio-available P along the elevation gradient of Shergyla Mountain, by analysis of phospholipid fatty acid” by Wang et al. Submitted for publication in PLOS ONE**

The study under review dealt with deciphering the relationship of P fractions and elevation gradients of mountain habitat of subalpine and alpine sites of Tibet, China on PLFA microbial community composition. The study determined the distribution pattern of PLFA biomarker microbial communities in relation to P availability prevailing across the gradient of mountain elevation. The understanding of this study may help in predicting the PLFA microbial communities and P availability while undertaking the management and rejuvenating the undisturbed soils/ecosystems. Overall study is nicely planned and executed and could be a good contribution in the science of microbial ecology. While going through the draft MS I noticed many shortcomings in relation of English language and typo errors which should be corrected while revising the MS legitimately.

**For the whole**

Title: revise the title as **Patterns of PLFA microbial communities were shaped by bio-available P along the elevation gradient of Shergyla Mountain”**

Adjust the space between reference and he last word of very sentence e.g., Line 32, 34 and so on.

Provide a brief description of microbial PLFAs (fatty acid signatures for bacteria, AMF, actinomycetes etc), stress indicator, total PLFA and their relationship with each other and environmental variables.

As microbial community analysis has only been performed through PLFA, no need to write PFA before microbial groups, such as AMF PLFA, Gram positive PLFA etc

Remove extra spaces from “( p < 0.05 )”

Findings of the study should be in past tense

There are numerous grammatical mistakes and I advise the authors to the help of native English speaker to improve the MS.

try to cite the references at the end of every sentence

**Abstract**

Line 10: You may change “The distribution pattern of microbial community in mountain is an important content of biodiversity research” to “The distribution pattern of microbial community in mountain is ~~an~~ important **for** /constitutes an integral component of biodiversity research.”

Line 15: What is meant by “many biomarkers”? Specify the biomarkers following the specified trend.

Line 21: “Microorganisms are strongly associated with P fractions” such kind of sentences are more suitable for introduction.

Line 22: “P stresses” or “P stress”?

**Introduction**:

Introduction needs restructuring.

Line 26: “Microbial diversity and community composition are important parameters in the study of soil microorganisms.” Either remove “microbial” or “microorganisms” and change the sentence accordingly.

Line 37: change “there is a large change in elevation” to “there is a considerable variation in the elevation”

Line 42: **Therefore,** it is necessary to know how

Line 45-46: Moreover, findings about the effects of elevation on microbial biogeography may **also** be applied to the ~~related study of the~~ **studies related to the** effects of latitude

Line 61: Change “and the factors that influence it were” to “influencing factors were”

Line 61-64: provide a brief discussion of their findings

Line 71-72: “between ~~patterns of~~ microbial community structure and soil P along

Line 72-73: “Thus, some important questions to investigate are ~~as follows~~”

Line 75: “The traditional method for studying soil microbial communities ~~has utilized~~ **utilizes** culture-dependent techniques that rely on the isolation of microbes from soil samples (e.g., microbial counts and membrane-filtration techniques).”

Line 77: **These methods** provide

Line 81: Write the full form of PLFA, since it is used for the first time

Line 89-90: No connection between the two paragraphs

Line 103: Check formatting After “and” “T” in “The” is capital

Line 110: Coniferous forest zone**s** are or Coniferous forest zone **is**

Line 111: change ‘is’ or ‘are’ accordingly

Line 120: Remove capitalisation. Authors may simply provide the reference instead of protocol name.

Line 152: Modify the sentence as “**The** marker for actinomycetes **was”**

Line 157: It should be **PLFA** not **PFLA**

Line 218: with **a** similar pH

Line 220-221: Change “soil moisture not display displayed” to “soil moisture did not display”

Line 221: Remove “mainly”

Line 234: three bioavailable P **forms**

Line 236: “**soil**” not **“soils”**

Line 237: Replace “great variations spatially” by “larger spatial variations”

Line 241: “were at very high levels” to” exhibited higher levels”

Line 244: Reframe the sentence “Microbial PLFA composition, ………different” You’ve used the word “microbial” three times

Line 247: “The total PLFA, the sum of the 44 fatty acids” such kind of sentences should be in the introduction

Line 250: “Bacterial PLFAs ~~comprised~~ **constituted** a large portion of the”

Line 257: Again, such sentences “The PLFA 16:1ω5c, which is representative of AMF” should be a part of introduction

Line 260: In place of “As the main component of bacterial population, like bacterial PLFAs, the PLFAs ascribed to Gram-positive bacteria also displayed a” write only Gram-positive bacteria. Everyone knows that this is a bacteria group

Line 262: In place of “For Gram-positive bacteria, their PLFAs,” write “For Gram-positive bacteria”

Line 267-268: replace “to presented all PLFA distribution” to “to **present** all PLFA distribution”

Line 278: such description “The proportion of individual PLFAs that were selected as biomarkers” is not required here

Line 278: also ~~changes~~ **changed**

Line 281: Fungal PLFAs ~~are~~ **were** significantly correlated

Line 283: PLFAs **were** all **strongly** correlate**d**”

Line 284: AMF **were negatively** correlate**d** with Resin-Pi

**Discussion**

Discussion needs improvement and restructuring

Line 287: Change the heading

Line 289-292: split the sentence into two parts

Line 294-300: Again, these sentences are appropriate for introduction

Line 301: or “**the variation** in the **microbial** PLFAs from ~~soil samples~~**”**

Line 310: Replace “Although our study and theirs” by “although the two studies”

Line 314: “Not coincidentally”..???

Line 35: “microbial biogeography was **primarily** controlled by edaphic variables and **fundamentally** differed……”

Line 320: “precipitation “**were”** the main factors affecting the distribution”

Line 322-23: Inappropriate sentence

Line 324: change the heading

Line 334: “also showed soil pH **as** the most important factor”

Line 334-335: “However, our study show**ed** that effects of soil pH **were** not”

Line 337: “of a very narrow pH range”

Line 337: “that the soil pH **was** and **did** not have”

Line 341: remove “much”

Line 343: remove “finding”

Line 346: “our results ~~are not so~~ **didn’t show any such trend**”

Line 346: replace “This mainly occurs for two reasons” by “Which could be attributed to/which could be due to/the reason for which could be” followed by the reason in points 1 and 2

Line 352: “the P ~~is~~ **was** likely to be the major limiting ~~nutrition~~ **nutrient**”

Line 354: remove “Especially” use “for instance”

Line 359: There **were** significantly negative

Line 371: remove “obviously”

Even after this point there are numerous grammatical mistakes. Authors are requested to check the and correct the same

**Conclusions**

**Line 389:** “which were often increasing **and showed** U-shaped and unimodal trends along…..”

Put space between supporting information

**Figures**

Fig : the details of fatty acids like cy/pre should only be provided in the introduction and methodology

Figure resolution needs improvement

**Tables**

Letters following the numbers should be in superscript
