# Supplementary material for: Patterns of microbial communities were shaped by bioavailable P along the elevation gradient of Shergyla Mountain, as determined by analysis of phospholipid fatty acids
Source: PLoS One. 2022 Jul 11;17(7):e0271101. doi: 10.1371/journal.pone.0271101 (PMC9273077; doi:10.1371/journal.pone.0271101)
Supplement: Attachment — Submitted filename: Response to Reviewers.docx [file pone.0271101.s003.docx]

Response to Reviewers

Dear Editor and Referee:

We have studied the valuable comments from you and referees carefully, and tried our best to revise the manuscript (Manuscript Number: **PONE-D-22-06081**). The point to point replies to the referees’ comments are listed as following:

**Reply to Journal Requirements:**

- **Comment 1:**

1. Please ensure that your manuscript meets PLOS ONE's style requirements, including those for file naming. The PLOS ONE style templates can be found at

https://journals.plos.org/plosone/s/file?id=wjVg/PLOSOne_formatting_sample_main_body.pdf and https://journals.plos.org/plosone/s/file?id=ba62/PLOSOne_formatting_sample_title_authors_affiliations.pdf

**Reply:**

According to the PLOS ONE style templates, our manuscript has been revised. We ensure that our manuscript meets PLOS ONE's style requirements.

- **Comment 2:**

2. We suggest you thoroughly copyedit your manuscript for language usage, spelling, and grammar. If you do not know anyone who can help you do this, you may wish to consider employing a professional scientific editing service.

Upon resubmission, please provide the following:

 The name of the colleague or the details of the professional service that edited your manuscript

A copy of your manuscript showing your changes by either highlighting them or using track changes (uploaded as a *supporting information* file)

**Reply:**

We have asked a language edit company (American Journal Experts, see the Editing Certificate in our covering letter) to help us to improve our manuscript. In its present form, we believe that the English quality is at the standard that is required for publication. In particular, in order to ensure correct grammar, the title is changed as follows: Patterns of microbial communities were shaped by bioavailable P along the elevation gradient of Shergyla Mountain, as determined by analysis of phospholipid fatty acids.

- **Comment 3:**

3. In your Methods section, please provide additional information regarding the permits you obtained to collect samples for the present study. Please ensure you have included the full name of the authority that approved the field site access and, if no permits were required, a brief statement explaining why.

**Reply:**

According to the comment, in your Methods section, we provided additional information regarding the permits we obtained to collect samples for the present study. See line 109-115 in the file " Revised Manuscript with Track Changes ".

Ethics Statement

All necessary permits were obtained for the described field studies. We conducted this study at the Shergyla Mountain, which is under the jurisdiction of the people's Government of Nyingchi City, Tibet Autonomous Region, people's Republic of China. We obtained permission from the people's Government of Nyingchi City to use the sample plots. Furthermore, our study did not harm the environment and did not involve endangered or protected species.

- **Comment 4:**

4. Thank you for stating the following in the Funding Section of your manuscript:

"This work was funded by intergovernmental cooperation in science, technology and innovation, and by the National Key Research and Development Program (2019YFE0194000), the National Natural Science Foundation of China (42077013)."

We note that you have provided funding information. However, funding information should not appear in the Funding section or other areas of your manuscript. We will only publish funding information present in the Funding Statement section of the online submission form.

Please remove any funding-related text from the manuscript and let us know how you would like to update your Funding Statement. Currently, your Funding Statement reads as follows:

"This work was funded by intergovernmental cooperation in science, technology and innovation, and by the National Key Research and Development Program (2019YFE0194000), the National Natural Science Foundation of China (42077013)."

Please include your amended statements within your cover letter; we will change the online submission form on your behalf.

**Reply:**

We removed any funding-related text from the manuscript. Our Funding Statement is as follows: This work was funded by intergovernmental cooperation in science, technology and innovation, and by the National Key Research and Development Program (2019YFE0194000), the National Natural Science Foundation of China (42077013).

Moreover, the Funding Statement is included within our cover letter.

- **Comment 5:**

5. Please include your full ethics statement in the ‘Methods’ section of your manuscript file. In your statement, please include the full name of the IRB or ethics committee who approved or waived your study, as well as whether or not you obtained informed written or verbal consent. If consent was waived for your study, please include this information in your statement as well..

**Reply:**

According to the comments, we provided the full ethics statement in the ‘Methods’ section of our manuscript file. See line 109-115 in the file "Revised Manuscript with Track Changes".

**Reply to the Additional Editor Comments**

- **Comment 1:**

Both reviewers specifically commented on the shortcomings in language. Please revise accordingly with the help of a native speaker.

**Reply:**

We have asked a language edit company (American Journal Experts, see the Editing Certificate in our covering letter) to help us to improve our manuscript. In its present form, we believe that the English quality is at the standard that is required for publication. The Editing Certificate is as follows:

**Reply to the Reviewers' comments:**

- **Comment 1:**

.1. Is the manuscript technically sound, and do the data support the conclusions?

The manuscript must describe a technically sound piece of scientific research with data that supports the conclusions. Experiments must have been conducted rigorously, with appropriate controls, replication, and sample sizes. The conclusions must be drawn appropriately based on the data presented.

Reviewer #1: Yes

Reviewer #2: Yes

**Reply:**

Thank the reviewers for their approval.

- **Comment 2:**

2. Has the statistical analysis been performed appropriately and rigorously?

Reviewer #1: Yes

Reviewer #2: Yes.

**Reply:**

Thank the reviewers for their approval.

- **Comment 3:**

3. Have the authors made all data underlying the findings in their manuscript fully available?

The PLOS Data policy requires authors to make all data underlying the findings described in their manuscript fully available without restriction, with rare exception (please refer to the Data Availability Statement in the manuscript PDF file). The data should be provided as part of the manuscript or its supporting information, or deposited to a public repository. For example, in addition to summary statistics, the data points behind means, medians and variance measures should be available. If there are restrictions on publicly sharing data—e.g. participant privacy or use of data from a third party—those must be specified.

Reviewer #1: Yes

Reviewer #2: Yes.

**Reply:**

Thank the reviewers for their approval.

- **Comment 4:**

4. Is the manuscript presented in an intelligible fashion and written in standard English?

PLOS ONE does not copyedit accepted manuscripts, so the language in submitted articles must be clear, correct, and unambiguous. Any typographical or grammatical errors should be corrected at revision, so please note any specific errors here.

Reviewer #1: Yes

Reviewer #2: Yes

**Reply:**

Thank the reviewers for their approval.

- **Comment 5:**

5. Review Comments to the Author

Please use the space provided to explain your answers to the questions above. You may also include additional comments for the author, including concerns about dual publication, research ethics, or publication ethics. (Please upload your review as an attachment if it exceeds 20,000 characters)

Reviewer #1: The manuscript presented a case study of microbial community variation along elevation gradient in south-eastern Tibetan Plateau. This is a nice work of soil microbial diversity, especially from the special alpine region.

There are some language mistakes including authors' affiliations.

Reviewer #2: The study under review dealt with deciphering the relationship of P fractions and elevation gradients of mountain habitat of subalpine and alpine sites of Tibet, China on PLFA microbial community composition. The study determined the distribution pattern of PLFA biomarker microbial communities in relation to P availability prevailing across the gradient of mountain elevation. The understanding of this study may help in predicting the PLFA microbial communities and P availability while undertaking the management and rejuvenating the undisturbed soils/ecosystems. Overall study is nicely planned and executed and could be a good contribution in the science of microbial ecology. While going through the draft MS I noticed many shortcomings in relation of English language and typo errors which should be corrected while revising the MS legitimately.

Please check the detailed comments submitted to editor.

**Reply:**

To improve the English language of our manuscript, we have asked a language edit company (American Journal Experts) to help us. In its present form, we believe that the English quality is at the standard that is required for publication.
